# Anticancer Effects of Wild Mountain *Mentha longifolia* Extract in Adrenocortical Tumor Cell Models

**DOI:** 10.3389/fphar.2019.01647

**Published:** 2020-02-10

**Authors:** Felicia Patti, Alessandro Palmioli, Sara Vitalini, Loris Bertazza, Marco Redaelli, Maira Zorzan, Beatrice Rubin, Caterina Mian, Cristina Bertolini, Maurizio Iacobone, Decio Armanini, Susi Barollo, Cristina Airoldi, Marcello Iriti, Raffaele Pezzani

**Affiliations:** ^1^Endocrinology Unit, Department of Medicine (DIMED), University of Padova, Padova, Italy; ^2^BioOrgNMR Lab, Department of Biotechnology and Biosciences, Università degli Studi di Milano Bicocca, Milan, Italy; ^3^Department of Agricultural and Environmental Sciences, Milan State University, Milano, Italy; ^4^AIROB, Associazione Italiana per la Ricerca Oncologica di Base, Padova, Italy; ^5^Venetian Institute for Molecular Science and Experimental Technologies, VIMSET, Liettoli di Campolongo Maggiore, Italy; ^6^Minimally Invasive Endocrine Surgery Unit, Department of Surgery, Oncology and Gastroenterology (DISCOG), University of Padova, Padova, Italy

**Keywords:** *Mentha longifolia*, mint, adrenocortical carcinoma, cell assay, cell models, phytotherapy

## Abstract

Mint [*Mentha longifolia* (L.) Hudson] is an aromatic plant that belongs to Lamiaceae family. It is traditionally used as herbal tea in Europe, Australia and North Africa and shows numerous pharmacological effects, such as spasmolytic, antioxidant, antimicrobial and anti-hemolytic. Recently, its antiproliferative role has been suggested in a small number of tumor cell models, but no data are available on adrenocortical carcinoma, a malignancy with a survival rate at 5 years of 20%–30% which frequently metastasize. This work aimed to study the effects of *Mentha longifolia* L. crude extract (ME) on two adrenocortical tumor cell models (H295R and SW13 cells). Chemical composition of ME was assessed by gas-chromatography/mass spectrometry and NMR spectroscopy analysis. Brine shrimp lethality assay showed ME effects at >0.5 µg/µl (p < 0.05). Cell viability and vitality were determined by MTT, SRB, and trypan blue assays in H295R and SW13 cells. The anti-proliferative effects of ME were more evident in SW13 cells at 72 h (ME > 0.5 µg/µl, p < 0.05). Combination of ME with mitotane (approved drug for adrenocortical carcinoma) seemed not to reinforce the efficacy of the herb. As control, human fibroblasts were treated with ME with no effect on cell viability. Clonogenic assay was concordant with previous cell viability tests (ME > 0.5 µg/µl, p < 0.05), while Wright staining demonstrated the presence of both necrotic and apoptotic cells. Cell cycle analysis showed a strong increase in subG0/G1 phase, related to cell death. Furthermore, MAPK and PI3k/Akt pathways were modulated by Western blot analysis when treating cells with ME alone or combined with mitotane. The crude methanolic extract of wild mountain mint can decrease cell viability, vitality and survival of adrenocortical tumor cell models, in particular of SW13 cells. These data show the potential anticancer effects of ME, still more work is needed to corroborate these findings.

## Introduction

Mint [*Mentha longifolia* (L.) Hudson] is a common aromatic herb easily found in the Mediterranean Region. It belongs to the Lamiaceae family and it is a wild perennial plant that can live at more than 1000m above the sea level. Extracts from *Mentha* species have been traditionally used for treating numerous and widespread diseases, such as indigestion, flatulence, irritable bowel syndrome, coughs, flu, nausea, gall-bladder, skin and respiratory infections, headache, and many others (Mimica-Dukic and Bozin, 2008). No apparent association seems to exist between the use of mint in humans and anti-proliferative ability and currently no clinical trial exists on the use of mint in cancer (Clinicaltrials.Gov, 2019). Nonetheless the potential effects on tumor cell lines of *Mentha* species were partially explored in preclinical models, as around twenty papers were in literature, with *Mentha piperita* (Linn.) the most studied species (Conforti et al., 2008; Hussain et al., 2010; Jain et al., 2011; Nedel et al., 2012; Al-Ali et al., 2013; Eissa et al., 2014; Sharma et al., 2014; Sun et al., 2014; Yang et al., 2017; Asemani et al., 2019). More specifically, *M. longifolia* L. used as methanolic/ethanolic/aqueous extracts or essential oils was investigated in different tumor cell models, demonstrating a strong cytotoxic activity (Hussain et al., 2010; Al-Ali et al., 2013; Eissa et al., 2014; Sharma et al., 2014; Asemani et al., 2019). No data is available for adrenocortical carcinoma cell models. Adrenocortical carcinoma is a rare neoplasia with a survival rate of 20%–30% at 5 years which frequently metastasizes (Pezzani, 2018). Main therapeutic option is surgery, whenever possible, while mitotane is an antineoplastic steroidogenesis inhibitor approved for the treatment of adrenocortical carcinoma. Mitotane can reduce tumor progression in 20%–25% of metastatic patients, but still it has heavy side effects (Stigliano et al., 2017). Given the lack of a concrete therapy for adrenocortical carcinoma, different pharmacological strategies were tried, though mint extract has been never evaluated in preclinical models of this rare malignancy. This work analyzed for the first time the effects of a crude methanolic extract of wild mountain mint (*M. longifolia*) for its anticancer potential in SW13 and H295R cells.

## Materials and Methods

### Plant Material

Wild mint [*Mentha longifolia* (L.) Hudson] has been harvested in uncultivated mountain land around the city of Vermiglio (TN—Italy, GPS position 46.268100, 10.654880), at about 1,600 m above sea level during summer season 2016. The plant was identified by one of authors (RP) and according to Pignatti (Pignatti, 2003). Leaves and flowers of mint were rapidly placed in sealed bags, until processing. A voucher specimen was deposited in the herbarium of the Department of Medicine, University of Padova, with specimen number 015ML.

### Crude Extract

Mint was shade dried and powdered using an electric blender. The powdered material was then extracted with methanol 80% for 24 h at 4°C. Subsequently the mixture was filtered through Whatman filter paper n°1 and resultant solution centrifuged to eliminate particulate matter. Methanol solvent was removed under reduced pressure using a rotary vacuum evaporator with controlled temperature (Rotavapor R-210, Flawil, Switzerland). The mint crude extract (ME) was successively weighed and dissolved in DMSO (w/v) to facilitate the solubility of the product. This ME+DMSO was used for all experiments, aliquoted and stored at -80°C. After thawing, each aliquot was used only once.

### GC/MS Analysis of Mint Extract

The chemical analysis was performed by re-solubilizing the dry extract in methanol followed by gas-chromatography/mass spectrometry (GC/MS) analysis. The splitless injection was in a DB-5ms, 30 m long capillary column, 0.25 mm in diameter, coated with a phenyl arylene film 0.25 um thick (Varian, Palo Alto, CA-USA). The MS was in full-scan mode in the m/z range between 20 and 600 amu. The GC temperature program was: 2 min at 45°C, a ramp of 5°C/min up to 280°C, holding at 280°C for 5 min, a ramp of 25°C/min up to 300°C, holding for 2 min (Cavaggioni et al., 2006). Identification of compounds was based on probability-matching of mass spectra using a computer library (NIST 2011, www.nist.gov, USA): results are to be intended as qualitative.

### NMR Analysis

The mint crude extract (ME) was suspended in phosphate buffer 10 mM pH 7.4 D_2_O and CD_3_OD (1:1) at a final concentration of 15mg/ml, sonicated (37kHz, 20min, Elmasonic P 30H, Elma Schmidbauer GmbH, Singen, Germany) and centrifuged (14,000rpm, 5min, 20°C, ScanSpeed 1730R Labogene, Lynge, Sweden). 3-(Trimethylsilyl)propionic-2,2,3,3-d_4_ acid sodium salt (TSP, final concentration 0.5mM) was added to the supernatant as internal reference for concentrations and chemical shift. The pH of each sample was verified with a microelectrode (Mettler Toledo, Columbus, OH, USA) and adjusted to 7.4 with NaOD or DCl. All pH values were corrected for the isotope effect. The acquisition temperature was 25°C. All spectra were acquired on an Avance III 600MHz NMR spectrometer (Bruker, Billerica, MA, USA) equipped with a QCI (^1^H, ^13^C, ^15^N/^31^P, and ^2^H) cryogenic probe. ^1^H NMR spectra were recorded with *noesygppr1d* pulse sequences (Bruker library) and 256 scans, spectral width 20ppm, relaxation delay 5s. They were processed with 0.3Hz line broadening, automatically phased and baseline corrected. Chemical shifts were internally calibrated to the TSP peak at 0.0ppm. The ^1^H,^1^H-TOCSY (Total Correlation SpectroscopY) spectra were acquired with 48 scans and 512 increments, a mixing time of 80ms and relaxation delay of 2s. ^1^H,^13^C-HSQC (Heteronuclear Single Quantum Coherence) spectra were acquired with 64 scans and 512 increments, relaxation delay 2s. The NMR data were processed using MestreNova 14.1.0 software (Mestrelab Research, Santiago de Compostela, Spain). Compound identification and assignments were done with the support of 2D NMR experiments and comparison with reported assignments. For metabolite quantification, the Simple Mixture Analysis (SMA) tool (Cobas et al., 2011) integrated in MestreNova software package was exploited to set a semi-automatic protocol for the identification and quantification of metabolites, by creating specific metabolite library. In this protocol, the GSD (global spectrum deconvolution) algorithm was employed to deconvolute the overlapping regions and to perform the absolute quantification of metabolites with resonances in crowded spectral areas, too. When possible, the concentration was calculated looking at the mean value of the different signals assigned to the same metabolite. Quantitative results were reported as mg of identified metabolite over one gram of crude mint extracts (mg/g ME).

### Materials and Reagent, Cell Cultures and Maintenance

Fetal bovine serum (FBS), 3-[4,5-dimethylthiazol-2-yl]-2,5 diphenyl tetrazolium bromide (MTT), propidium iodide, mitotane, and all other chemicals were purchased from Sigma Aldrich (Italy). DMEM-F12, 0.05% trypsin-EDTA, insulin, transferrin, selenium, and antibiotics were from Thermo Scientific (Milan, Italy). Adrenocortical tumor cells (H295R and SW13 cells) were obtained from the American Type Culture Collection (ATCC, Rockwille, MD). SW13 cells have a doubling time of about 24 h, H295R cells show a >48–72 h doubling time: this difference has been considered in cell experiments (Wang and Rainey, 2012). Furthermore, human fibroblasts derived from a donor were used as normal diploid human cells. Written informed consent to the collection and use of these cells for research purposes was obtained. All cells were cultured as previously described (Mariniello et al., 2012).

### Brine Shrimp Lethality Bioassay

Brine shrimp lethality bioassay (BSLB) was performed for investigating the cytotoxicity of ME as previously described (Rubin et al., 2018). The extract was tested at 10, 5, 2, 1, 0.5 μg/μl. Brine shrimps (*Artemia salina*) were hatched in a round shaped vessel (1 L), with sterile artificial seawater (24 g sodium chloride, 5 g magnesium chloride, 4 g sodium sulfate, 1.14 g calcium chloride, 0.7 g potassium chloride, 0.17 g sodium bicarbonate in 1 L of distilled water and pH 8.2 with sodium hydroxide) and continuous oxygen supply. After 48  h, the active nauplii were transferred to a 96-well plate and used for the study with ME at 0.0625, 0.125, 0.25, 0.5, 1, 2, 10 μg/μl. Twenty nauplii were added to each well and kept them in room temperature (24-26°C) for 24  h under light. After that time, dead larvae were counted. The mean percentage mortality was plotted against the logarithm of concentrations and the LC_50_ (concentration that kills fifty percent of the nauplii) was determined using the probit analysis described by Finney (Finney, 1949) as well as linear regression equation using the software “Microsoft Excel 2010”. The experiments were performed in quadruplicates and repeated three times.

### MTT Assay and IC_50_

SW13 and H295R cells were plated in 96-well tissue culture micro titer plates at a density of 5×10^3^ cells/well as described elsewhere. Cells were treated with ME at 0.0625, 0.125, 0.25, 0.5, 1, 2 μg/μl for 24 and 72 h following the method previously described (Pezzani et al., 2014).

The Inhibitory Concentration 50 (IC_50_, defined as 50% of the inhibitory effect on cell viability) was calculated (Bertazza et al., 2015). Also mitotane was used at 10 μM alone (the concentration in cell lines resembling the quantity of mitotane circulating in human serum for the treatment if adrenocortical carcinoma) or combined with ME 0.5 μg/μl. Each analysis was performed in quadruplicate and repeated three times.

### Sulforhodamine B Assay

SW13 and H295R cells plated in were trypsinized and seeded in 96-well tissue culture micro titer plates at a density of 5×10^3^ cells/well as already reported (Bertazza et al., 2018). After 24 h incubation, cells were treated with ME 0.0625, 0.125, 0.25, 0.5, 1, 2 μg/μl for 24 and 72 h. Negative control for the experiment was 0.1% DMSO in DMEM/F12 medium. Cells were then fixed with 20 μl of 50% trichloroacetic acid and incubated for 1 h at 4°C. After washing, cells were stained with 50 μl of 0.4% (w/v) SRB solution. After 20 min of incubation at room temperature, plates were washed with 1% acetic acid. Bound SRB dye was then solubilized by adding 100 μl of Tris base solution. Absorbance was read at 540 nm OD on Victor 3 (Perkin Elmer). Each analysis was performed in quadruplicate and repeated three times.

### Trypan Blue Dye Exclusion Method

Trypan blue dye exclusion method was used to assess cell viability after treatment with ME at 0.0625, 0.125, 0.25, 0.5, 1, 2, 10 μg/μl. The assay was performed at 24 and 72 h. At the end of treatment, cells were collected by trypsinization, centrifuged and the cell pellet was resuspended in 1 ml of PBS. Next, 10 µl of the resulting cell suspension was admixed with 10 µl of Trypan blue (0.4% in PBS). The numbers of non-stained viable cells (NSt cells) and stained dead cells (St cells) were counted using a hemocytometer. Cell viability was then calculated by the following formula: Viability % = (NSt cells)/(St cells + NSt cells) x 100. Each analysis was performed quadruplicate and repeated three times.

### Assessment of Cell Morphology by Wright’s Staining

Cells were cultured on coverslips and then processed with ME at 0.0625, 0.125, 0.25, 0.5, 1, 2 μg/μl. H295R cells were treated for 72 h, while SW13 cells for 24 h, following the method previously described (Barollo et al., 2014). Also mitotane was used at 10 μM alone (not shown) or combined with ME (B1 = ME 0.25 μg/μl + mitotane 10μM, C1 = ME 0.5 μg/μl + mitotane 10μM, D1 = ME 1 μg/μl + mitotane 10μM). Cell morphology was evaluated by light microscopy at x400 magnification. Cells were counted by two independent experts (MR and RP) and at least 600 cells were counted for every experiment in at least 10 different fields and each experiment was repeated twice.

### Cell Cycle Analysis

Cells were plated at a density of 1x10^6^ cells/well and treated with ME (0.5 μg/μl), mitotane 10 μM, and their combination, trypsinized and harvested by centrifugation. SW13 cells were incubated for 72 h, while H295R cells were incubated for 96 h, as already described (Mariniello et al., 2012). Cells were resuspended in ice-cold PBS and fixed in 70% ice-cold ethanol followed by an overnight incubation at -20°C. After washing, cells were stained with propidium iodide solution (50 mg/ml PI, 10 mg/ml RNaseA) and incubated for 1 h at 37°C in darkness. Analysis was carried out by using the CytoFLEX 13/3 and data were analyzed by CytExpert software (Beckman Coulter, Fullerton, CA, USA). Each experiment was repeated three times.

### Clonogenic Cell Survival Assay

Cells were seeded in 96-well plates at a low density (1,000 cells per well) as previously described (Rubin et al., 2018). Briefly cells were incubated overnight in 0.1% FBS and then treated with ME (0.0625, 0.125, 0.25, 0.5, 1 μg/μl for SW13 cells) or mitotane 10 μM or their combination (mitotane+ME 0.25 μg/μl) for 24 h (SW13 cells) and 72 h (H295R cells). Then cell medium was replaced with free medium and cell cultured for 1 week (SW13 cells) or 2 weeks (H295R cells). Cells were then fixed and stained with crystal violet. Only colonies >50 cells were counted. Each experiment was performed in triplicate and repeated two times.

### Western Blot Analysis

Cells were treated with ME (0.5 μg/μl) at 24 and 72 h as previously described (Pezzani et al., 2014). Mitotane 10 μM was used alone or combined with ME. Briefly, cells were treated with lysis buffer (40 mM HEPES, 120 mM sodium chloride, 10 mM sodium pyrophosphate, 10 mM sodium glycerophosphate, 1 mM EDTA, 50 mM sodium fluoride, 0.5 mM sodium orthovanadate, and 1% Triton X-100) containing a protease inhibitor cocktail (Sigma–Aldrich) for 1 h at 4°C. Then proteins were extracted and centrifuged collecting supernatant. Total proteins were quantified, separated by SDS/PAGE, electro-blotted onto nitrocellulose membranes and saturated in 5% fat-free dried milk. Membranes were incubated overnight with primary antibodies and then incubated with secondary ones (Sigma-Aldrich). Immunoreactivity was detected by LiCor Odyssey infrared scanner and bands intensity quantified by Image Studio 3.1 analytical software (LiCor, Lincoln, NE, USA). The primary antibodies were: anti-Erk1/2, anti-phospho-Erk1/2 (Thr202/Tyr204), anti-Akt, anti-phospho-Akt (Ser473), all diluted 1:1,000 (Cell Signaling, Danvers, MA, USA), and anti-β-actin diluted 1:5,000 (Sigma-Aldrich). All experiments were performed in triplicate.

### Statistical Analysis

Statistical analysis was performed using GraphPad Prism 6 software (GraphPad Software, Inc., San Diego, CA). Nonlinear regression and sigmoidal dose-response curves were used to calculate the IC_50_ values for each cell line. Data comparisons were performed using the two-tailed Student's t test and Kruskal-Wallis analysis followed by Dunn's post-test. Mann-Whitney test was applied for band quantification statistical analysis. *p* < 0.05 was considered statistically significant.

## Results

### Mint Extract GC/MS and NMR Analysis

GC/MS analysis returned a complex profile that was processed for qualitative analysis. **Table 1** reports the identified compounds. The chromatographic profile is compatible with a vegetal extract.

**Table 1:** List of the identified compounds *via* GC/MS analysis of mint crude extract. The identification is qualified by the column Match % NIST11 representing NIST11 library spectrum similarity (qualification under 35% were not reported).

Peak N	RT	Library/ID	CAS	Match % NIST11
1	5,79	N-Acetylglycine	000543-24-8	47
2	5,99	Methyl pyruvate	000600-22-6	72
3	6,38	2-Methylpyrazine	000109-08-0	86
4	6,56	Furfural	000098-01-1	87
5	6,70	2-Furanmethanol	000098-00-0	96
6	7,18	2,5-Diethyltetrahydrofuran	041239-48-9	74
7	7,80	1-methyl-azetidine	004923-79-9	53
8	8,27	1-Nonene	000124-11-8	35
9	10,62	5-Hydroxyuridine	000957-77-7	38
10	11,62	N-Methyl-1,3-propanediamine	006291-84-5	38
11	11,81	4,5-Diamino-1,2-dihydropyrimidin-2-one	023899-73-2	47
12	13,37	Catechol	000120-80-9	87
13	13,74	2,3-Dihydrobenzofuran	000496-16-2	93
14	13,99	2,6-Di-tert-butylnaphthalene	003905-64-4	72
15	14,13	Hydroquinone	000123-31-9	64
16	14,27	2-Hexyn-1-ol	000764-60-3	49
17	14,93	Thymol	000089-83-8	93
18	15,25	Diosphenol	000490-03-9	58
19	15,37	2,2-Dimethylcyclohexanone	001193-47-1	38
20	15,51	2-Methoxy-4-vinylphenol	007786-61-0	94
21	16,15	Eugenol	000097-53-0	98
22	16,64	6-Isopropyl-benzothiazol-2-ylamine	032895-14-0	43
23	17,38	4-Methoxypyridine	000620-08-6	35
24	17,63	Dracorhodin	000643-56-1	64
25	17,81	Salicylaldehyde hydrazone	003291-00-7	45
26	18,02	2-Chloro-3-hydrazinopyrazine	063286-28-2	49
27	18,45	2-Chlorothiophenol	006320-03-2	52
28	18,87	2-Methyltetrahydrothiophene	001795-09-1	43
29	20,45	Methyl alpha-D-glucopyranoside	000097-30-3	38
30	21,44	1-Octanamine	000111-86-4	38
31	22,74	2,3,4-Trimethoxybenzoic acid	000573-11-5	43
32	23,17	Loliolide	005989-02-6	89
33	23,61	1,2,3,4,5-Cyclopentanepentol	056772-25-9	83
34	23,87	Undecanoic acid	000112-37-8	35
35	24,18	Methyl 14-methylpentadecanoate	005129-60-2	92
36	24,70	n-Hexadecanoic acid	000057-10-3	99
37	26,45	Phytol	000150-86-7	98
38	26,63	Methyl stearate	000112-61-8	95
39	26,93	Linolenic acid	000463-40-1	99
40	27,10	Octadecanoic acid	000057-11-4	99
41	27,36	Civetone	000542-46-1	44
42	30,33	2-Benzylcyclopentanecarboxylic acid	073742-04-8	41
43	30,94	Diethylamine	000109-89-7	50
44	32,09	Glyceryl palmitate	000542-44-9	90
45	36,48	Glyceryl monostearate	000123-94-4	91

**Figure 1:**
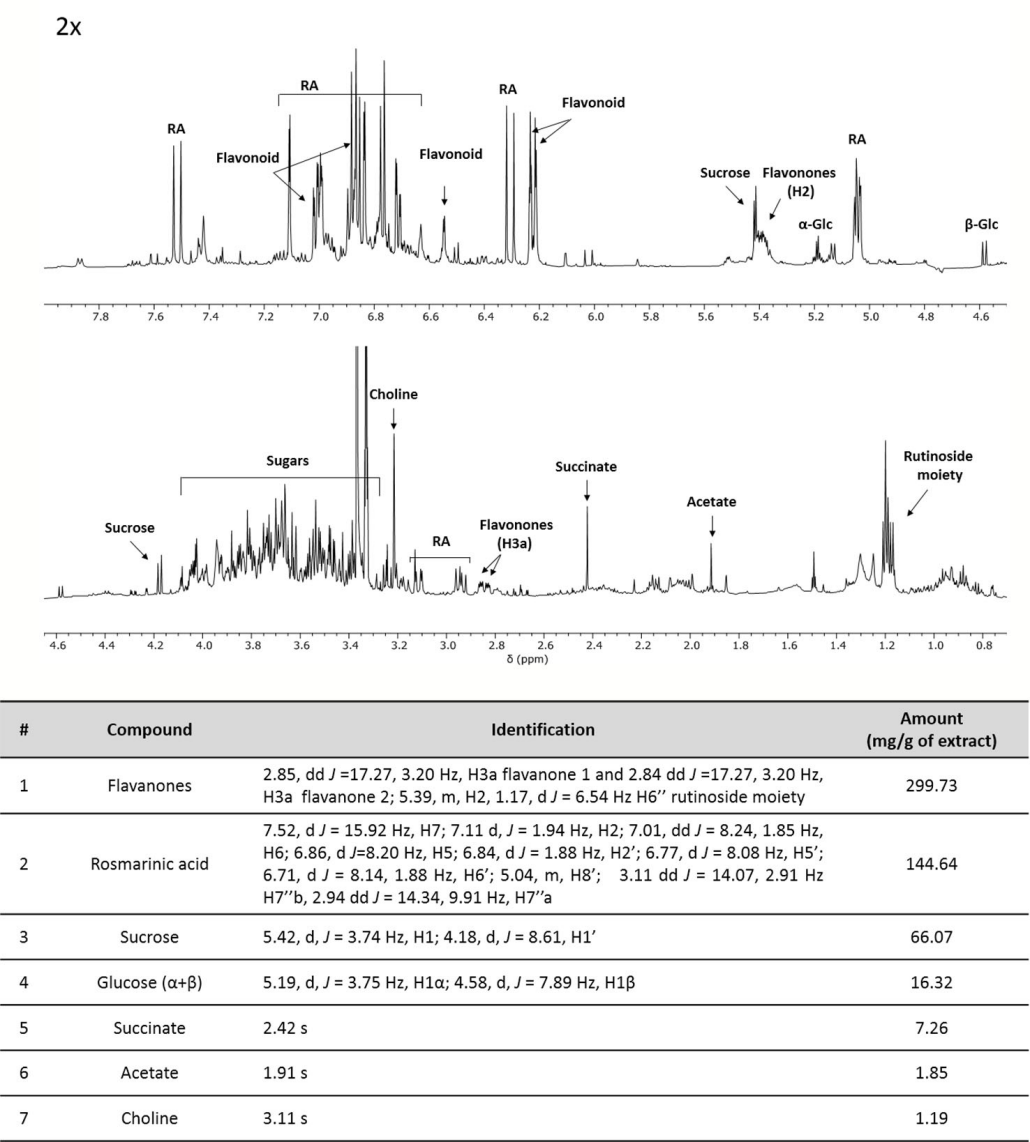
^1^H-NMR profile and of crude mint extract (ME) dissolved in PB 10 mM D_2_O: MeOD (1:1) at a concentration of 15 mg/ml (TSP 0.5 mM, pH 7.4, 25°C). Table containing metabolites' concentrations and detailed peak identification was reported below. RA, rosmarinic acid; Glc, glucose.

In addition, ME was characterized by NMR spectroscopy data exploited for primary and secondary metabolites identification and quantification (including non-ionizable and non-volatile compounds) following the approach developed for the analysis of complex plant extracts (Amigoni et al., 2017; Palmioli et al., 2017; Ciaramelli et al., 2018; Ciaramelli et al., 2019; Palmioli et al., 2019). The identification of metabolites was based on mono and bidimensional NMR spectra analysis and is in agreement with data from previous literature (Brahmi et al., 2015; Fotakis et al., 2016). After the manual identification of compounds, a specific library was built using the Simple Mixture Analysis (SMA) tool implemented in the MestReNova 14.1 software. SMA allows for the simultaneous quantification of all metabolites contained in a complex mixture. The library developed with this approach is available as.exp files (Palmioli and Airoldi, 2019). Overall, ^1^H NMR profile (**Figure 1**) reveals a fair amount of rosmarinic acid (144.64 mg/g ME) and at least two major undistinguished flavanones rutinoside (299 mg/g ME), supposedly eriocitrin and hesperidin. In addition, sucrose (66.07 mg/g ME), α- and β-glucose (16.32 mg/g ME), succinate (7.26 mg/g ME), acetate (1.85 mg/g ME) and choline (1.19 mg/g ME) were found.

Moreover, antioxidant and antiradical properties of ME extract can be found in **Supplementary File 1**.

### BSLB

**Figure 2:**
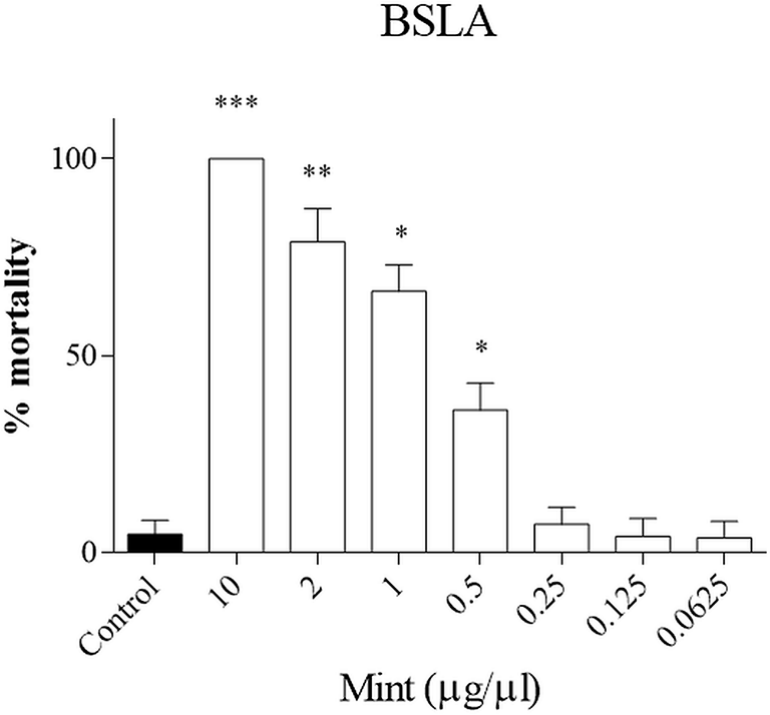
Brine shrimp lethality assay (BSLA). ME was tested at 10, 2, 1, 0.5, 0.25, 0.125, 0.0625 μg/μl, while control was untreated. Cytotoxic effect of ME on shrimp nauplii was examined after 24 h as per protocol. Calculated LC50 was 0.70 ± 0.29 μg/μl. Experiments performed in quadruplicates and repeated three times. *p < 0.05; **p < 0.005; ***p < 0.001.

First the toxicity of the plant was evaluated in a small animal model, the brine shrimp lethality assay (BSLA). We showed that only higher concentrations of ME were toxic for survival of nauplii (ME > 0.5 µg/µl, p < 0.05), while lower concentrations did not influence survival (**Figure 2**). The LD_50_ calculated with the probit analysis was 0.70 ± 0.29 μg/μl.

### Cell Viability Analysis

**Figure 3:**
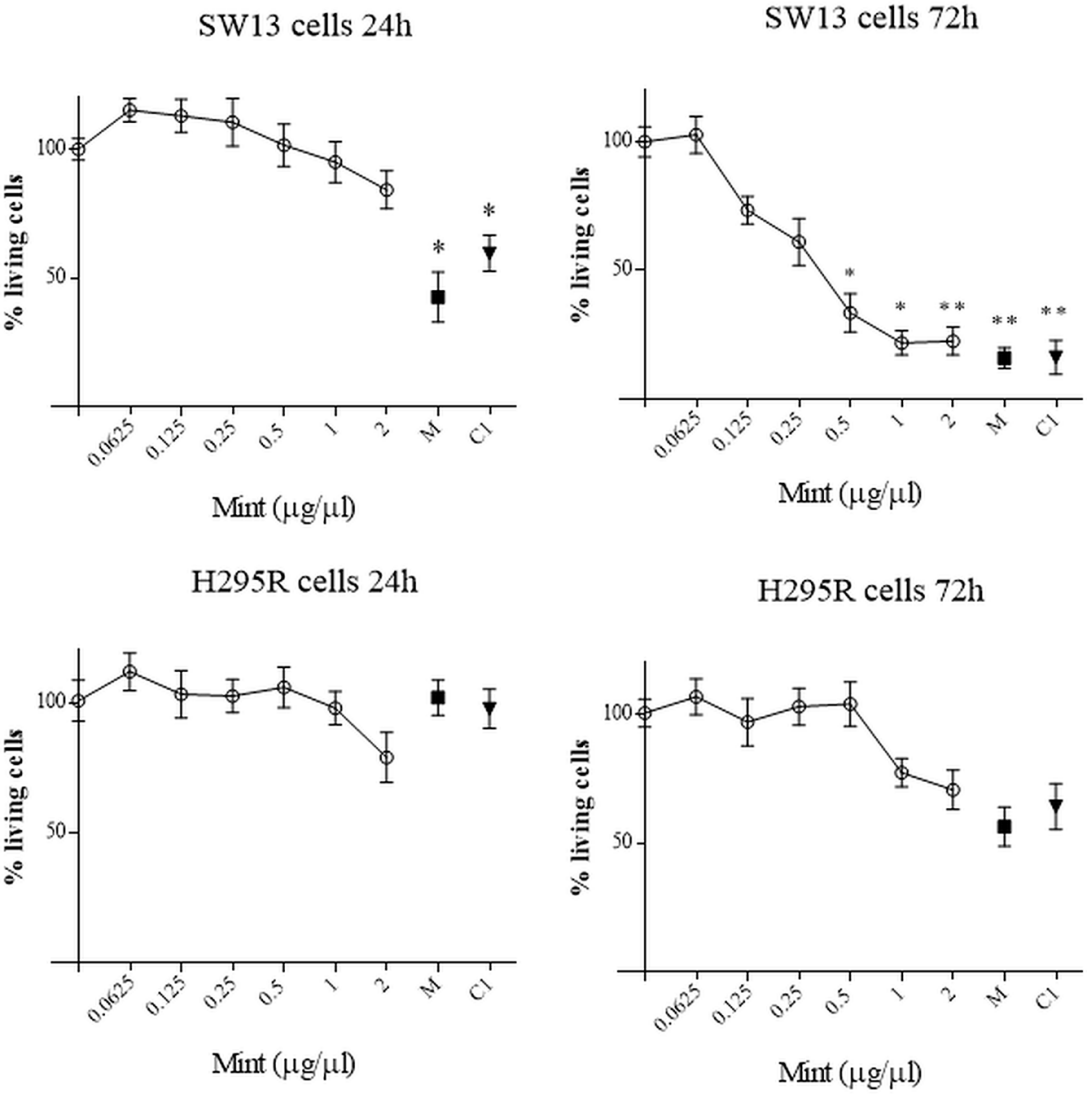
MTT assay on SW13 and H295R cells at 24 and 72 h, respectively. M: mitotane 10 μM. C1: mitotane 10 μM + ME 0.5 μg/μl. The results are expressed as a percentage of control (100%). Treatment *vs* control: *p < 0.05; **p < 0.005. Each analysis was performed in quadruplicate and repeated three times.

The effects of ME were investigated in SW13 and H295R cells at different time and concentration. MTT test showed that ME induced a concentration and time-dependent inhibition of mitochondrial reduction activity especially in SW13 cells at higher concentrations and at 72 h (ME > 0.5 µg/µl, p < 0.05), with an IC_50_ of 0.34 ± 0.11 μg/μl at 72 h (**Figure 3**). H295R cells did not reached an IC_50_ value, while we observed a trend decrease at higher concentrations (**Figure 3**). Moreover, the use of mitotane, the approved drug for adrenocortical cancer, in combination with ME did not show a reinforced effect in both cell lines. We also performed SRB assay to test cytotoxic activity of ME and we showed a reduction in total cell proteins similar to MTT results, with a reduction of SW13 cells proteins at 72 h (ME > 0.5 µg/µl, p < 0.05) (**Supplementary Figure 1**). Accordingly, by trypan blue dye exclusion method we measured the number of viable cells and we noticed a decrease of cell number more evident in SW13 cells at 72 h (ME > 0.5 µg/µl, p < 0.05) (**Supplementary Figure 2**). Of note that ME showed no toxicity for human fibroblasts (**Supplementary Figure 3**), in line with BSLA results (**Figure 2**).

### Assessment of Cell Morphology

**Figure 4:**
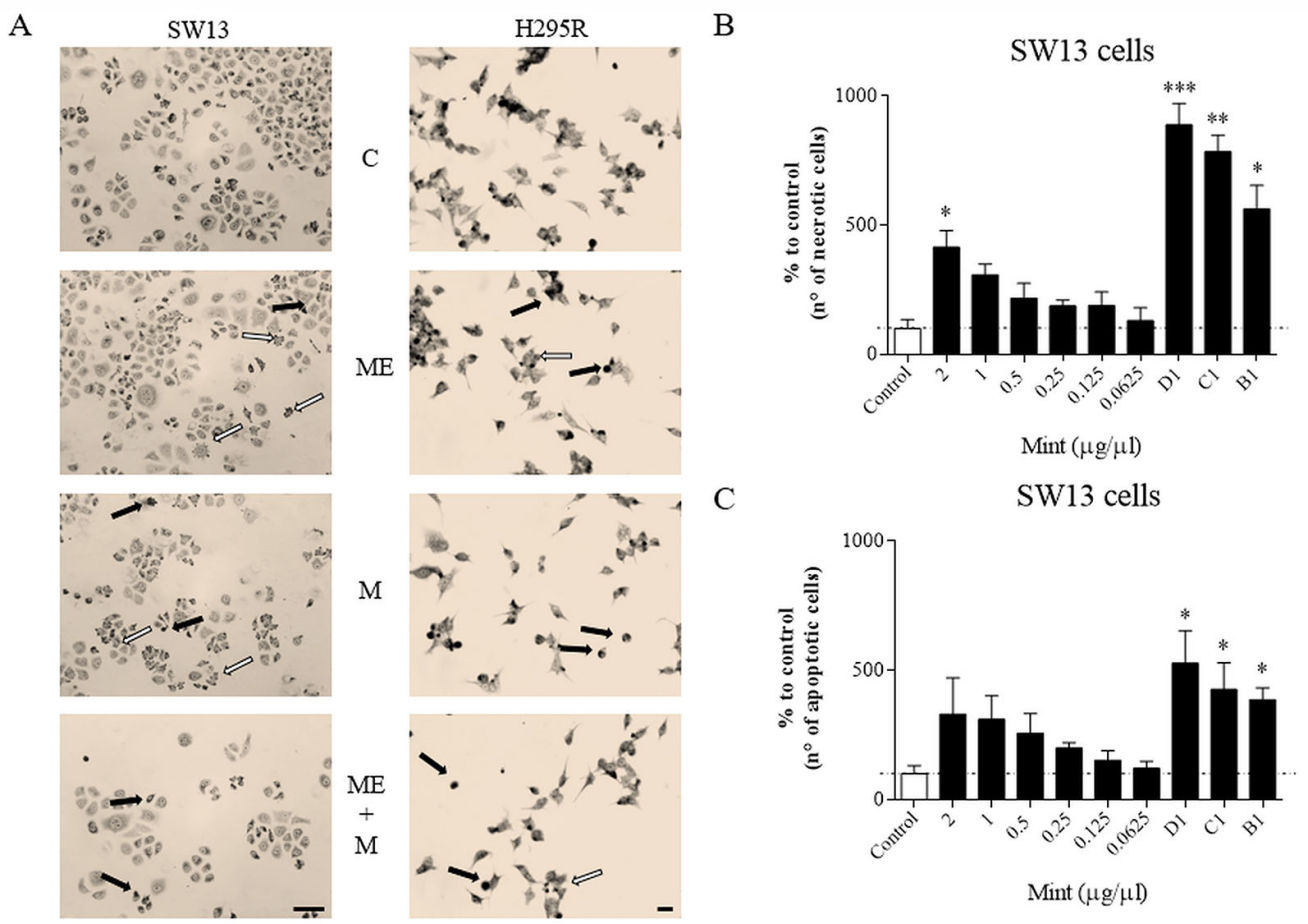
Cells morphology evaluated by Wright's staining method in SW13 and H295R cells at 24 and 72 h, respectively. **(A)** representative pictures of SW13 and H295R cells. The arrows show apoptotic (white) or necrotic cells (black). **(B**, **C)** number of counted cells for SW13 cell line. B1: ME 0.25 μg/μl + mitotane 10μM. C1: ME 0.5 μg/μl + mitotane 10μM. D1: ME 1 μg/μl + mitotane 10μM. Treatment *vs* control: *p < 0.05; **p < 0.005; ***p < 0.001. At least 600 cells were counted for every experiment in 10 different fields and each experiment was repeated twice.

Wright's staining investigated apoptosis (detectable as blebbing, cell shrinkage, nuclear fragmentation, condensation of chromatin) and necrosis (detectable as membrane breakdown and cell disruption) by light microscopy. Morphological changes were shown for SW13 and H295R cells (**Figure 4A**). Necrotic cells were increased at higher concentrations (p < 0.05 only for ME = 2 µg/µl in SW13 cells) and when combined with mitotane (p < 0.05 only for B1, C1, D1 in SW13 cells), while the number of apoptotic cells were not dissimilar to control, with the exception of combination regimen (p < 0.05 only for B1, C1, D1 in SW13 cells) (**Figures 4B–C**). H295R cells did not show any statistical difference between apoptotic or necrotic cells, except for higher ME concentrations associated with mitotane (**Supplementary Figure 4**).

### Cell Cycle Distribution

**Figure 5:**
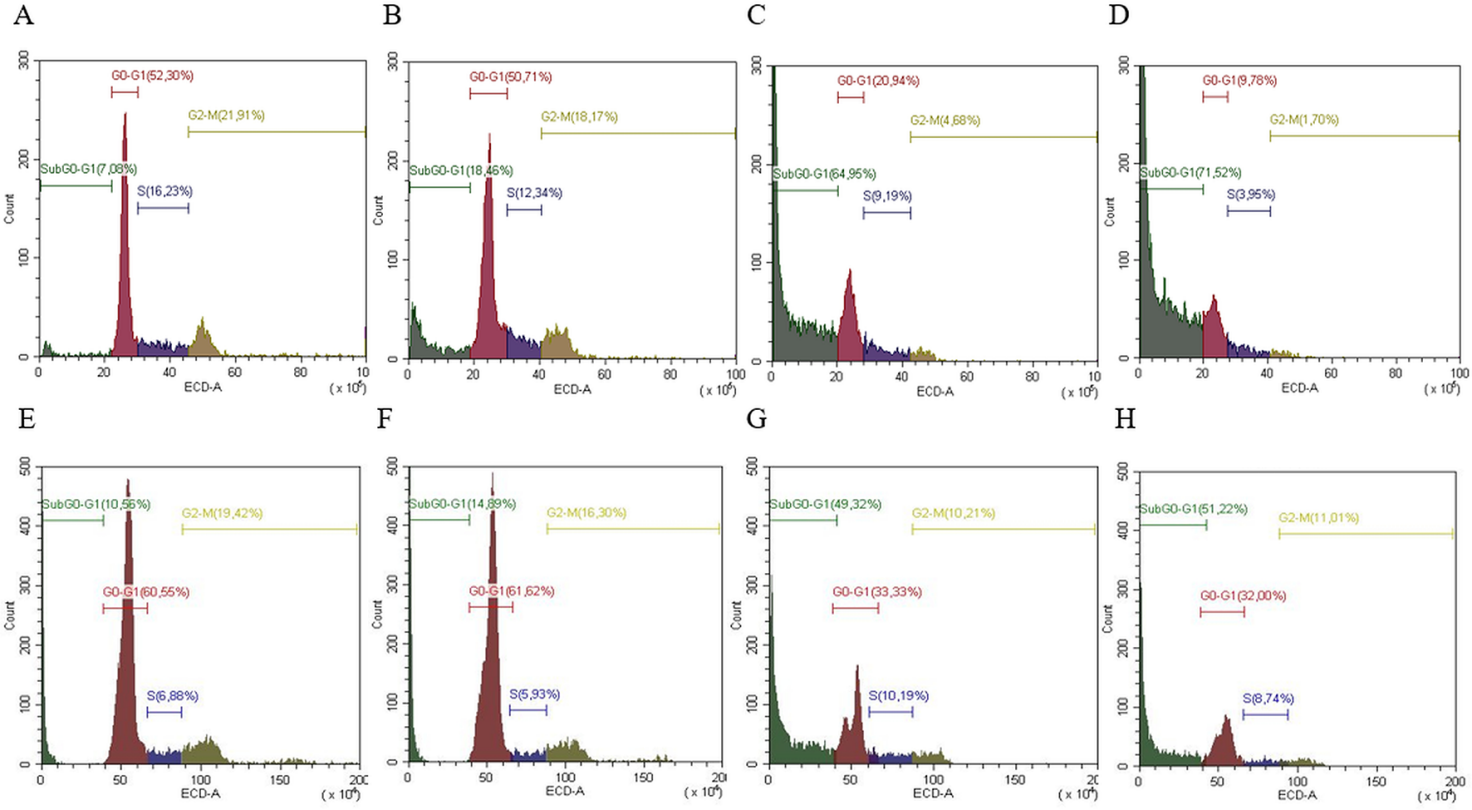
Representative cell cycle analysis for SW13 and H295R cells. SW13 cells treated at 24 h: **(A)** Control; **(B)** ME; **(C)** mitotane; **(D)** mitotane+ME. H295R cells treated at 72 h: **(E)** Control; **(F)** ME; **(G)** mitotane; **(H)** mitotane+ME. Experiments performed in triplicate.

Cell cycle analysis was evaluated in both cell lines (SW13 and H295R cells) in order to understand if there was any modulation of cell cycle distribution. In SW13 cells, an abundant increase in subG0/G1 phase if compared to control was observed in mitotane or ME+mitotane, while ME alone did not induce an ample subG0/G1 increase. A concomitant decrease of G0/G1 and G2/M phases were shown when ME alone or ME+mitotane were used (**Figures 5A–D**). In H295R cells, comparable results were observed (**Figures 5E–H**).

### Clonogenic Cell Survival Assay

**Figure 6:**
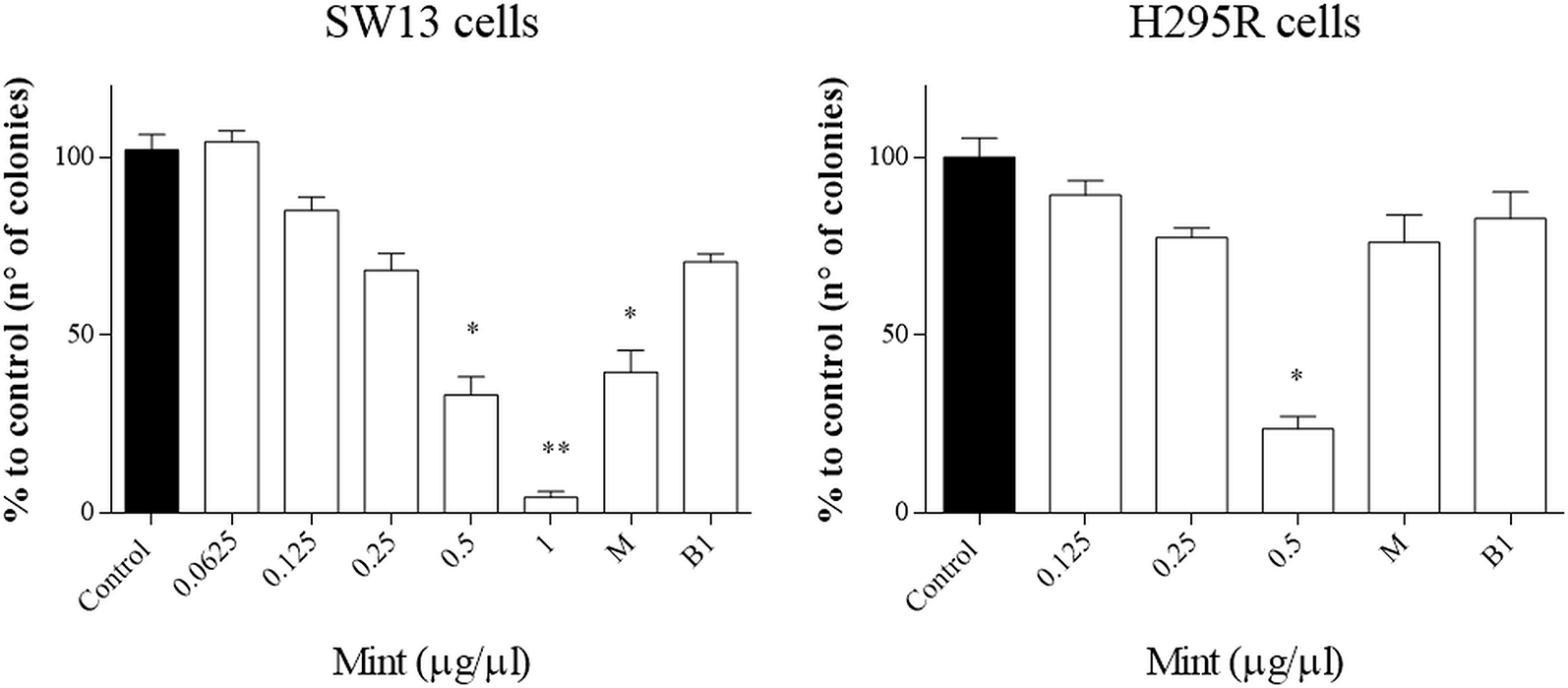
Representative clonogenic assay for SW13 and H295R cells treated at 24 and 72 h, respectively. **(A)** SW13 cells. **(B)** H295R cells. M: mitotane. B1: mitotane 10 μM + ME 0.25 μg/μl. Treatment vs control: *p < 0.05. Each experiment was performed in triplicate and repeated two times **p < 0.005.

The clonogenic survival assay is a test based on the ability of a single cell to grow into a colony, a hallmark of cancer and can analyze stemness-like activity of the cells. The effects of ME showed a decrease in colony number at higher concentrations (>0.5 µg/µl, p < 0.05) and in both cell lines, but not when combined with mitotane (**Figure 6**). Only mitotane alone in SW13 showed a statistically decrease of cell colony number (mitotane 10 µM, p < 0.05).

### Western Blot Analysis

**Figure 7:**
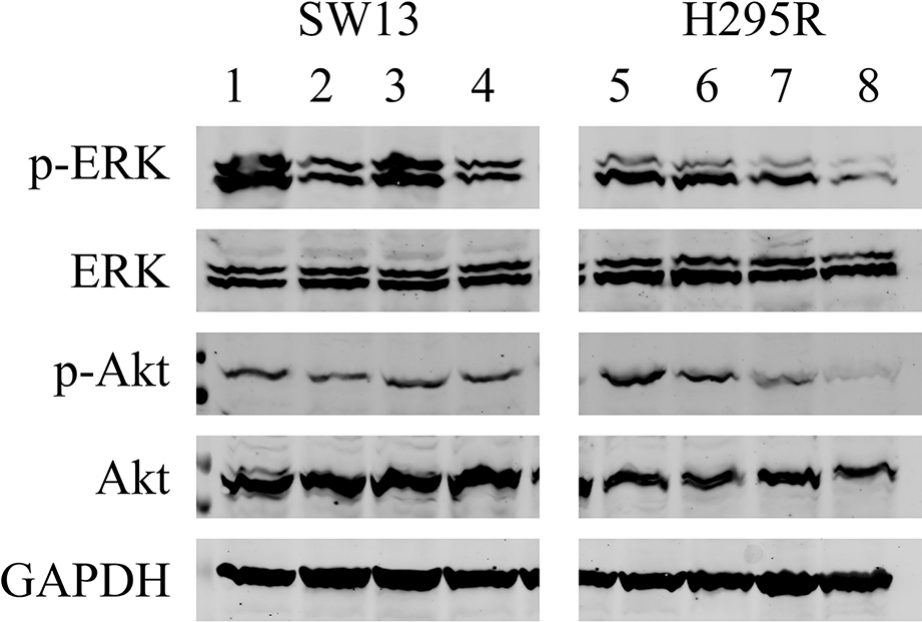
Representative Western blot analysis for SW13 and H295R cells. Lines 1 and 5: untreated control. Lines 2 and 6: 0.5 μg/μl ME. Lines 3 and 7: mitotane 10 μM. Lines 4 and 8: ME 0.5 μg/μl + mitotane 10 μM. Experiments performed in triplicate.

We analyzed the effects of ME alone or combined with mitotane on both cell lines (**Figure 7**). In SW13 cells ME partially reduced phospho-Erk1/2 (line 2) if compared to untreated control (line 1), while no effect was perceivable for phospho-Akt (lines 2 and 4). Mitotane alone produced a similar reactivity for phospho-Erk1/2 (line 3) as the control (line 1), while mitotane combined with ME decreased phospho-Erk1/2 signal (line 4). In H295R cells, ME did not modify phospho-Erk1/2 or phospho-Akt (line 6) while ME+mitotane diminished phosphorylated antibodies (line 8). Mitotane alone slightly reduced both phosphorylated antibodies (line 8).

## Discussion

This research work analyzed for the first time a mint extract (*M. longifolia*) on adrenocortical tumor cell lines. This plant was collected in a wild high mountain terrain and the extract (ME) was tested for its potential multiple properties in biochemical and biological models. First, we qualitatively and quantitatively evaluated the ME by GC/MS and NMR. It was mainly characterized by the presence of rosmarinic acid and two flavanones. The anti-proliferative activity of these compounds was already reported. In general, high content of rosmarinic acid and other phenolic compounds in aromatic plant extracts appeared to be, at least partially, responsible for their cytotoxicity (Ćebović et al., 2018; Martins-Gomes et al., 2018; Altay et al., 2019). Moreover, different flavanones showed anti-proliferative and apoptotic activities (Abd El-Hafeez et al., 2019; Koosha et al., 2019). Specifically, hesperidin and eriocitrin are known as anti-cancer agents (Birsu Cincin et al., 2015; Julius et al., 2017; Cao et al., 2019). Recently, hesperidin was found able to potentiate, along with two other natural compounds, the effect of tamoxifen against the breast tumour (Khamis et al., 2018) while eriocitrin to reduce the incidence of oral squamous cell carcinoma induced in hamsters (Baldasquin-Caceres et al., 2014).

Subsequently, as we needed to assess the ME potency in a complex living system, we investigated the extract in a vertebrate model of toxicity: the BSLA is an easy and very informative test used to evaluate the potential harmfulness of a novel extract (Freires et al., 2017). Only ME concentrations > 0.5 µg/µl resulted critically toxic in the animal model and thus conceivable useful in cancer (**Figure 1**). Taken into account this outcome, we successively evaluated ME in cell models of adrenocortical tumors. In particular, we used the 2 most common models, SW13 and H295R cells. Of note that SW13 cells are a depot in the adrenal of a primary lung cancer, while H295R cells derive from a female affected by an adrenocortical carcinoma (Wang and Rainey, 2012) and this fact can explain the different behavior of the 2 cell lines. Viability assays showed a strong effect of ME in SW13 cells at 72 h, when ME concentrations were >0.5 µg/µl, independently to mitotane (**Figure 2**, **Supplementary Figure 1** and **Figure 2**). Even if using different concentrations and type of extracts of *M. longifolia*, similar results were observed in breast, astrocytoma, prostate, cervical, T-cell lymphoblast, urinary bladder carcinoma, colon, pancreatic, kidney epithelial carcinomas (Hussain et al., 2010; Jain et al., 2011; Al-Ali et al., 2013; Eissa et al., 2014; Sharma et al., 2014). Association of ME and mitotane did not show a synergic effect: in the best case they showed an additive result, but more probably they interacted with an antagonistic action (Blaszczyk et al., 2018). It is not possible at this point to understand which compounds in the plant phytocomplex interfere with mitotane. This needs to be deepen with more experimental sets and, first and foremost, should be confirmed with a more specific test, such as the combination index. Even if ME should not be associated with mitotane, ME alone can still be used in preclinical models. Indeed, ME could impact on the ability of cells to duplicate and grow up. Effectively SW13 cells have a duplication time greater than H295R cells (approximately double). This hypothesis seems in accordance with cell cycle results, where an increase in subG0/G1 phase suggested cell death, again independently by mitotane (**Figure 4**). Moreover, this result implied an unchanged cell cycle distribution in adrenocortical cell lines when treated by ME, mitotane or their combination. Cell death process can be roughly subdivided into 2 different fates, apoptosis or necrosis. By morphological evaluation, we noted a major increase of necrotic cells than apoptotic in SW13 model (**Figure 3**), probably underlining a genotoxic mechanism, but this assumption needs to be further addressed (Roos et al., 2016). ME can induce a strong cell death mechanism, suggested not only by cell cycle analysis, but also by cell morphology assay and by viability assays. Similarly other works showed anticancer property of *M. longifolia* extract, even if in different cell models (Al-Ali et al., 2013; Asemani et al., 2019). Additionally, in support of ME effects on cells, the clonogenic assay showed that the extract could impede the stemness-like activity of both adrenocortical cell models (**Figure 5**), with a concentration >0.5 µg/µl. Moreover, when mitotane was added to ME, the capacity to reduce the number of colonies was not significant in both cell lines, again emphasizing as the combination of the drug (mitotane) with the extract seemed not be beneficial.

Western blot analysis showed that in SW13 cells, ME could switch off MAPK signaling (**Figure 6**, lines 2 and 4, independently of mitotane combination) and consequently decrease cell proliferation and growth without modifying PI3k/Akt pathway (Lawrence et al., 2008). Differently in H295R cells, ME seemed not to impact on both MAPK and PI3k/Akt signaling pathways. This fact suggests that any specific effect of the extract could reside in other pathways not analyzed in this work (Xia et al., 2014; Alimbetov et al., 2018). As expected, mitotane alone or combined with ME decreased the reactivity of Erk1/2 and Akt in H295R cells, as already demonstrated (Bertazza et al., 2019). Moreover the combination of ME+mitotane in both cell lines advocated that the anticancer effects of the compounds was not antagonistic nor synergic but rather additive (Tang et al., 2015; Pezzani et al., 2019), even if more research is needed to corroborate this hypothesis.

This research work analyzed the properties of a methanolic extract of wild mountain *M. longifolia*. Its anticancer activities were explored in a vertebrate and for the first time in adrenocortical tumor cell models. These results suggest that ME can positively impact on preclinical systems and lay the foundation to explore ME in higher organisms.

## Data Availability Statement

All datasets generated for this study are included in the article/**Supplementary Material**.

## Ethics Statement

Ethical review and approval was not required for the study on human participants in accordance with the local legislation and institutional requirements. The patients/participants provided their written informed consent to participate in this study. Ethical review and approval was not required for the animal study because we used brine shrimp animals that do not need ethical approval.

## Author Contributions

Conceptualization: RP. Methodology: FP, SV, LB, SB, MR, MZ, BR, RP, AP, CA. Formal and Statistical Analysis: MR, CM, CB, MI, RP. Writing—Original Draft Preparation: RP, SV, MR. Writing—Review and Editing: FP, SV, CM, CB, MI, DA, MIr, RP. Supervision: SV, DA, SB, MIr, RP.

## Conflict of Interest

The authors declare that the research was conducted in the absence of any commercial or financial relationships that could be construed as a potential conflict of interest.

## Supplementary Material

Supplementary Figure 1 Cell viability on SW13 and H295R cells at 24 and 72h tested by SRB assay. The results are expressed as a percentage of control (100%). Treatment vs control: *p > 0.05; **p > 0.005. Each analysis was performed in quadruplicate and repeated 3 times.

Supplementary Figure 2 Cell viability on SW13 and H295R cells at 24 and 72h tested by trypan blue dye exclusion assay. The results are expressed as a percentage of control (100%). Treatment vs control: *p > 0.05; **p > 0.005. Each analysis was performed in quadruplicate and repeated 3 times.

Supplementary Figure 3 MTT test on fibroblasts. Cells were treated with different ME concentrations at 24h. The results are expressed as a percentage of control (100%). Treatment vs control: *p > 0.05; **p > 0.005. Each analysis was performed in quadruplicate and repeated 3 times.

Supplementary Figure 4 Cells morphology evaluated by Wright’s staining method in H295R at 72h. **(A)** representative pictures of SW13. The arrows show apoptotic (white) or necrotic cells (black). **(B**, **C)** number of counted cells. Treatment vs control: *p > 0.05; **p > 0.005; ***p > 0.001. At least 600 cells were counted for every experiment in 10 different fields and each experiment was repeated twice.

Data Sheet 1 Antioxidant activity assessment of mint extract, including total polyphenol content, ABTS^•+^ and DPPH radical-scavenging assays.
