# Supplementary figures and images for: Anticancer Effects of Wild Mountain Mentha longifolia Extract in Adrenocortical Tumor Cell Models
Source: Front Pharmacol. 2020 Feb 10;10:1647. doi: 10.3389/fphar.2019.01647 (PMC7025550; doi:10.3389/fphar.2019.01647)

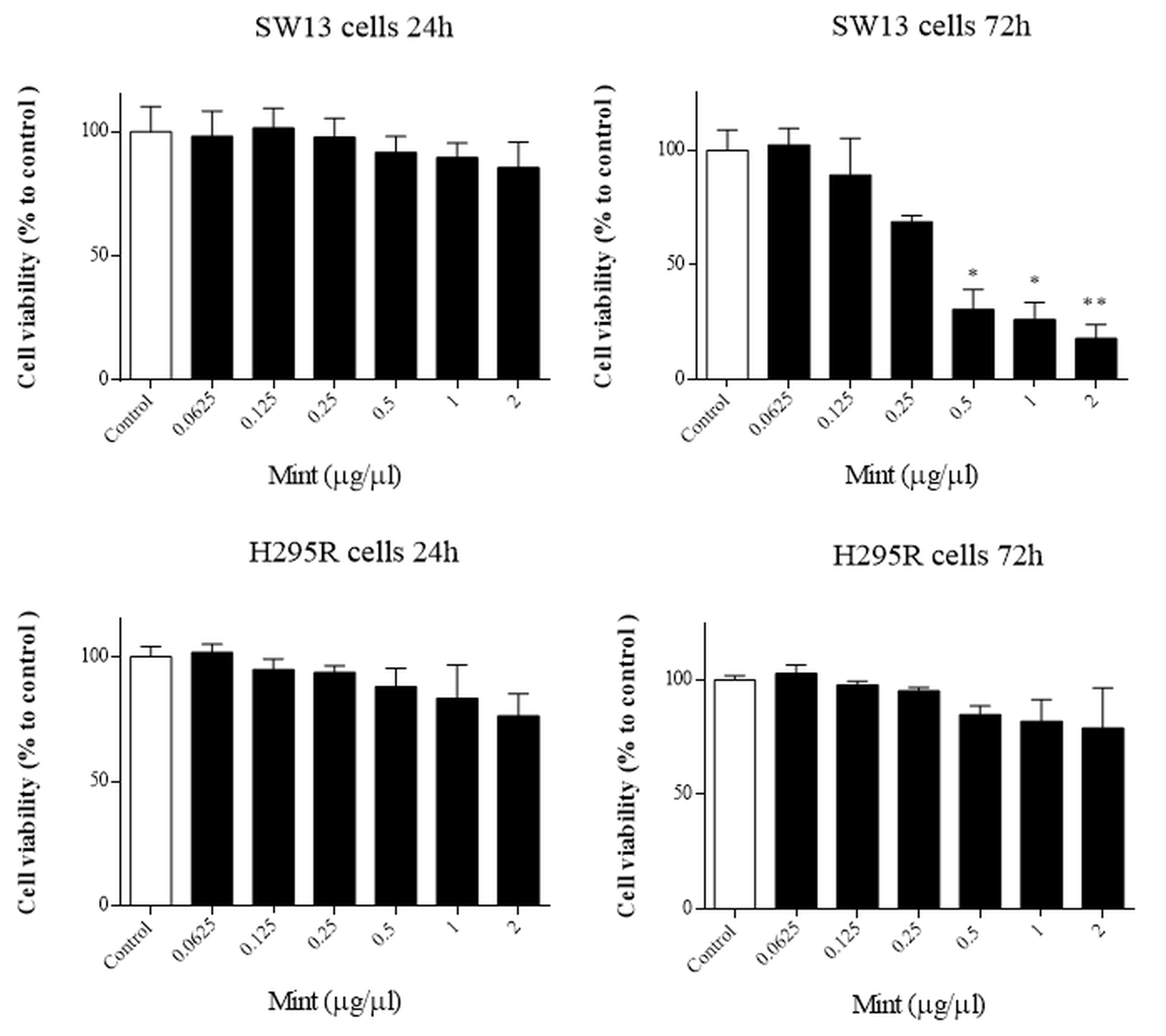

Supplement: Supplementary Figure 1 — Cell viability on SW13 and H295R cells at 24 and 72h tested by SRB assay. The results are expressed as a percentage of control (100%). Treatment vs control: *p > 0.05; **p > 0.005. Each analysis was performed in quadruplicate and repeated 3 times. [file Image_1.jpeg]

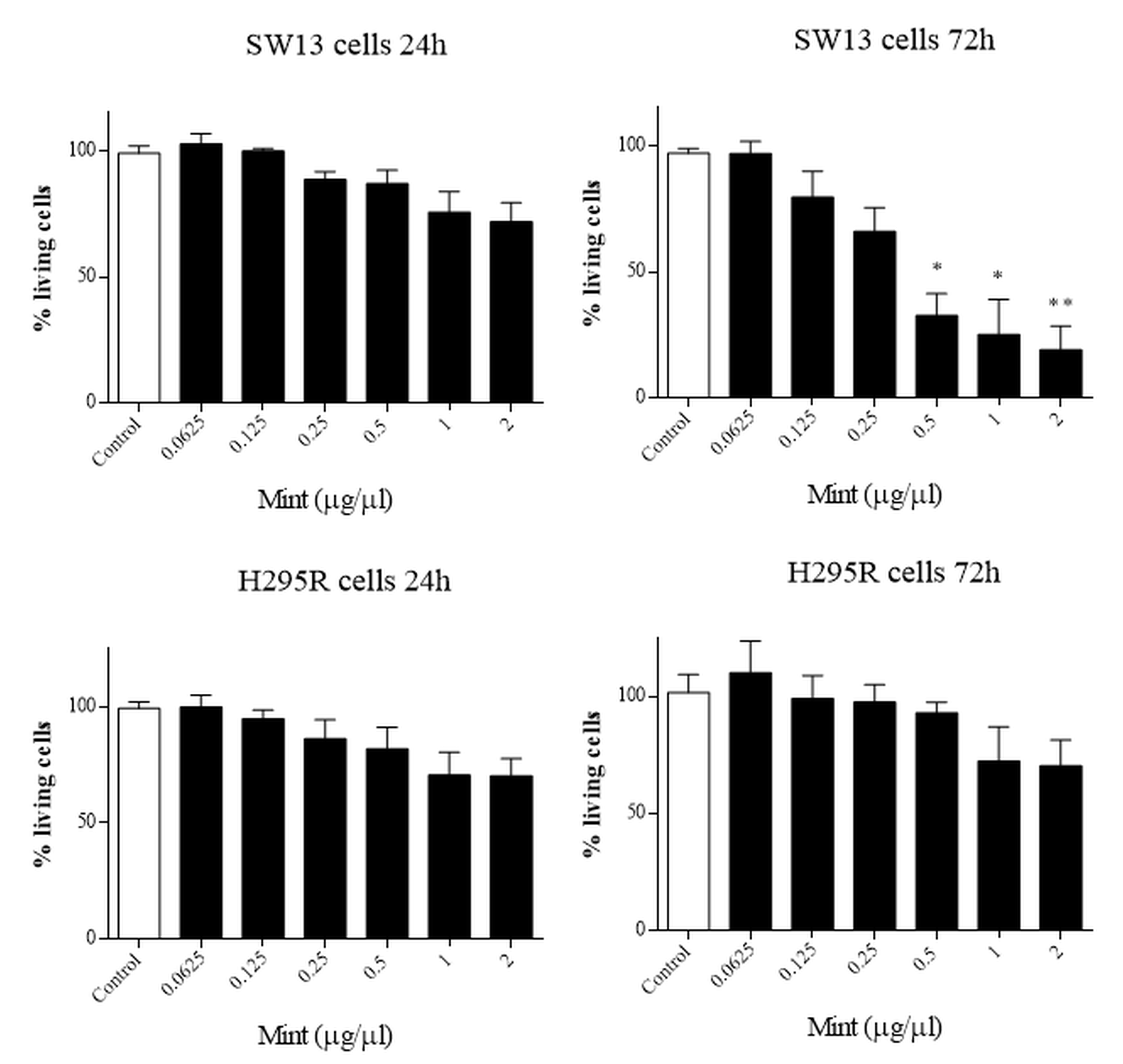

Supplement: Supplementary Figure 2 — Cell viability on SW13 and H295R cells at 24 and 72h tested by trypan blue dye exclusion assay. The results are expressed as a percentage of control (100%). Treatment vs control: *p > 0.05; **p > 0.005. Each analysis was performed in quadruplicate and repeated 3 times. [file Image_2.jpeg]

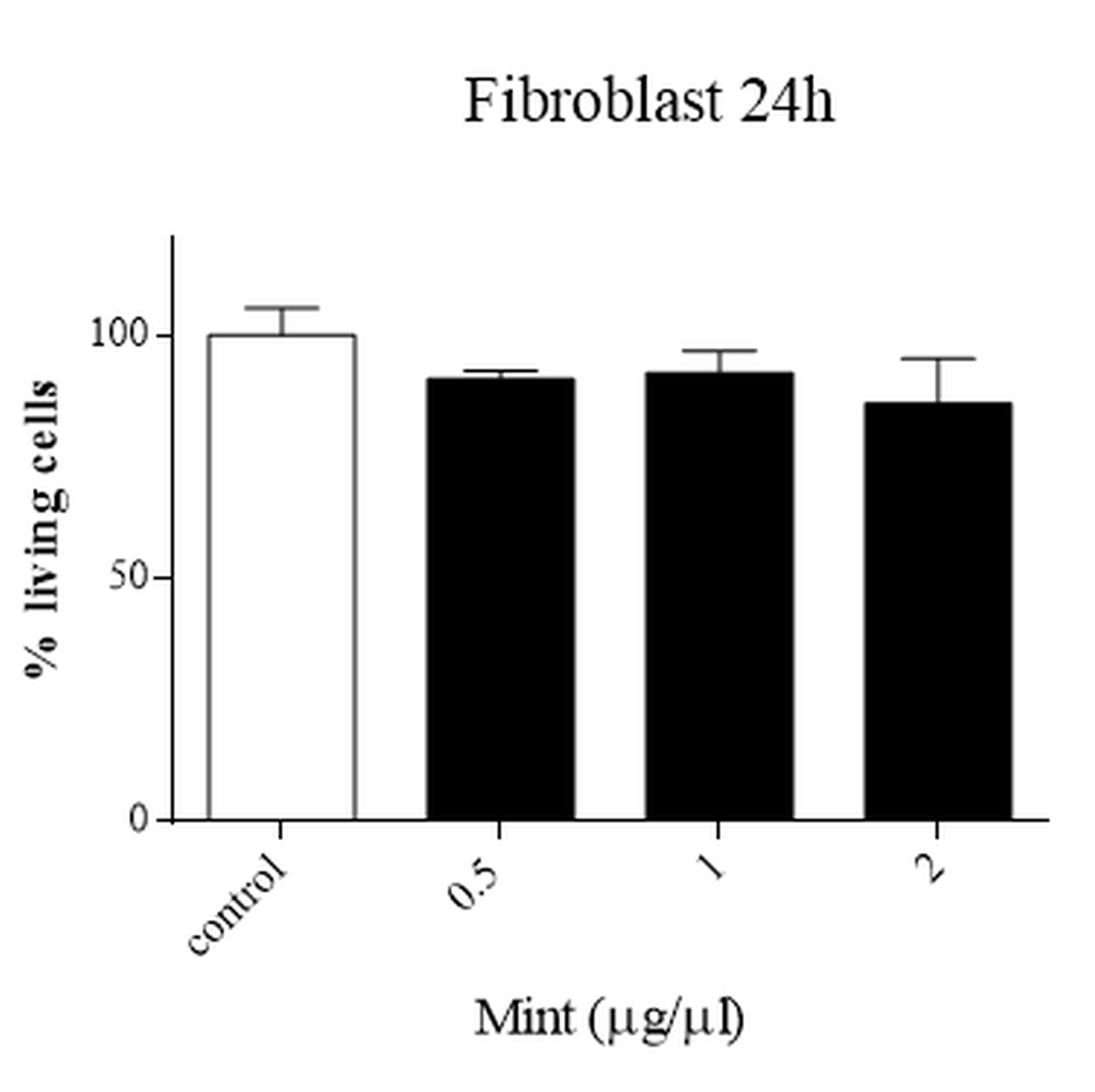

Supplement: Supplementary Figure 3 — MTT test on fibroblasts. Cells were treated with different ME concentrations at 24h. The results are expressed as a percentage of control (100%). Treatment vs control: *p > 0.05; **p > 0.005. Each analysis was performed in quadruplicate and repeated 3 times. [file Image_3.jpeg]

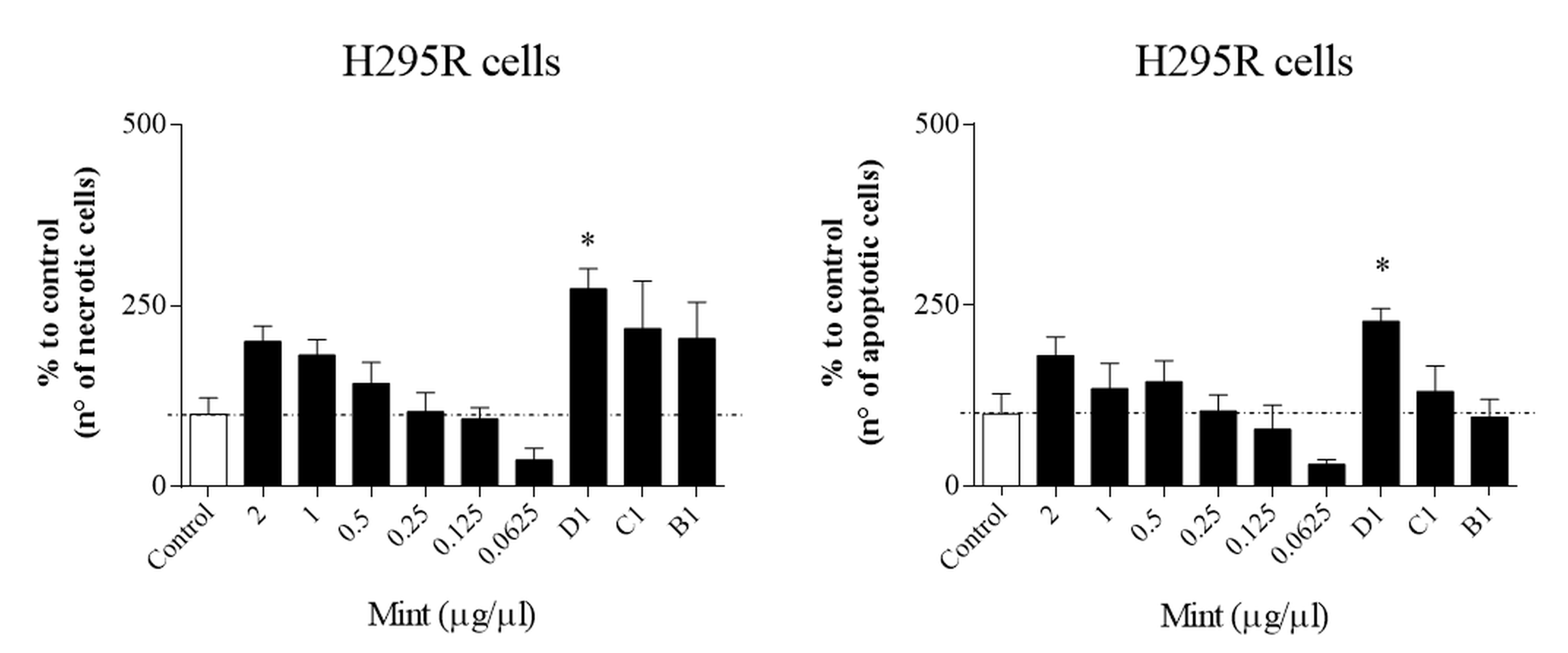

Supplement: Supplementary Figure 4 — Cells morphology evaluated by Wright’s staining method in H295R at 72h. (A) representative pictures of SW13. The arrows show apoptotic (white) or necrotic cells (black). (B, C) number of counted cells. Treatment vs control: *p > 0.05; **p > 0.005; ***p > 0.001. At least 600 cells were counted for every experiment in 10 different fields and each experiment was repeated twice. [file Image_4.jpeg]
