# Supplementary material for: Anticancer Effects of Wild Mountain Mentha longifolia Extract in Adrenocortical Tumor Cell Models
Source: Front Pharmacol. 2020 Feb 10;10:1647. doi: 10.3389/fphar.2019.01647 (PMC7025550; doi:10.3389/fphar.2019.01647)
Supplement: Data Sheet 1 — Antioxidant activity assessment of mint extract, including total polyphenol content, ABTS•+ and DPPH radical-scavenging assays. [file DataSheet_1.docx]

**Supplementary File1**

**Materials and Methods**

*Determination of total polyphenol content*

Total polyphenol content was measured by the Folin-Ciocalteu colorimetric assay ([Visioli et al., 1995](#_ENREF_30)). Brieﬂy, an aliquot of the sample was added to 50 μL of Folin-Ciocalteu reagent. The solutions were mixed and allowed to stand for 3 min. Next, 100 μL of a saturated sodium carbonate solution and distilled water to ﬁnal volume of 2.5 mL were added. After 1 h of incubation, in the dark, at room temperature, the absorbance was read at 725 nm. Polyphenol quantiﬁcation was based on a standard curve (0.1– 500 mg/L, R^2^ = 0.9999) of gallic acid, and results were expressed as mg of gallic acid equivalents per g of extract (mg GAE/g).

*ABTS^∙+^ radical-scavenging assay*

The 2,2’-azino-bis(3-ethylbenzothiazoline-6-sulfonic acid) (ABTS**^∙^**^+^) radical cation-scavenging capacity was determined according to Vitalini et al. (2016). The ABTS**^∙^**^+^ radical cation was produced by reacting 7 mM ABTS with 2.45 mM potassium persulfate (final concentration) and maintaining the mixture in the dark at room temperature for, at least, 6 h before use. The ABTS**^∙^**^+^ solution was diluted with ethanol to an absorbance of 0.7 (± 0.02) at 734 nm and equilibrated at 30 °C. Ten μL of the sample, ethanol (negative control) and standard solution of the synthetic antioxidant 6-hydroxy-2,5,7,8-tetramethychroman-2-carboxylic acid (Trolox, positive control) were mixed for 30 s with 1 mL of diluted ABTS**^∙^**^+^ solution. Their absorbance was read at 734 nm, at room temperature, 50 s after the initial mixing. The results are reported as Trolox equivalent antioxidant capacity (TEAC, μmol eq Trolox g^-1^).

*DPPH^.^ radical-scavenging assay*

The 2,2-diphenyl-picryl hydrazyl (DPPH·) radical-scavenging capacity was performed following ([Vitalini et al., 2016](#_ENREF_31)). In brief, aliquots the sample, at ﬁve different concentrations (from 1–100 µM), were added to 0.07 mM MeOH solution of DPPH· free radical reaching a ﬁnal volume of 2 mL. After a reaction time of 30 min in the dark at room temperature, the decrease in absorbance was measured at 517 nm. The IC_50_ was calculated with Prism®4 (GraphPad Software Inc., La Jolla, CA, USA).

**Results**

*Total polyphenol content, ABTS^∙+^ and DPPH^.^ radical-scavenging activity*

The total polyphenol content of ME was 190.7 mg GAE/g. When the polyphenol concentration was referred to the aerial parts, the content was found equal to mg 86.1 mg GAE/g dry weight and was comparable to that reported in some earlier studies ([Hajlaoui, 2009](#_ENREF_13);[Iqbal et al., 2013](#_ENREF_15)) or higher than detected in other ones ([Spiridon, 2011](#_ENREF_26);[Patonay, 2017](#_ENREF_20)),. In terms of antiradical actions, the extract exhibited a rather high degree of activity. In particular, it was able to scavenge the stable radical DPPH∙ with an IC_50_ value of 5.45±0.01 µM while the ABTS^∙+^ inhibition measured in ME corresponded to a TEAC value of 174.7±4.2 µmol eq Trolox/g in accordance with previous data ([Hajlaoui, 2009](#_ENREF_13); [Patonay K, 2017](#_ENREF_20); [Bahadori, 2018](#_ENREF_3)).

**References**

Bahadori, M.B., Zengin, G., Bahadori, S., Dinparast, L., Movahhedin, N. (2018). Phenolic composition and functional properties of wild mint (*Mentha longifolia* var. *calliantha* (Stapf) Briq.). . *International J. Food Prop.* 21**,** 183-193.

Hajlaoui, H., Trabelsi, N., Noumi, E., Snoussi, M., Fallah, H., Ksouri, R., Bakhrouf, A. (2009). Biological activities of the essential oils and methanol extract of tow cultivated mint species (Mentha longifolia and Mentha pulegium) used in the Tunisian folkloric medicine. . *World J. Microbiol. Biotechnol.* 25**,** 2227-2238.

Iqbal, T., Hussain, A.I., Chatha, S.A., Naqvi, S.A., and Bokhari, T.H. (2013). Antioxidant Activity and Volatile and Phenolic Profiles of Essential Oil and Different Extracts of Wild Mint (Mentha longifolia) from the Pakistani Flora. *J Anal Methods Chem* 2013**,** 536490.

Muranyi, Z,. Pénzesné Konya, E. (2017). Polyphenols in northern Hungarian *Mentha longifolia* (L.) L. treated with ultrasonic extraction for potential oenological use. *Turkish Journal of Agriculture and Forestry* 41**,** 208-217.

Patonay, K., Korozs, M., Muranyi, Z., and Pénzesné Konya, E. (2017). Polyphenols in northern Hungarian Mentha longifolia (L.) L. treated with ultrasonic extraction for potential oenological use. . *Turk. J. Agric. For.* 41.

Spiridon, I., Bodirlau, R., and Teaca, C.A. (2011). Total phenolic content and antioxidant activity of plants used in traditional Romanian herbal medicine. *Cent. Eur. J. Biol.* 6**,** 388-396.

Vitalini, S., Madeo, M., Tava, A., Iriti, M., Vallone, L., Avato, P., Cocuzza, C.E., Simonetti, P., and Argentieri, M.P. (2016). Chemical Profile, Antioxidant and Antibacterial Activities of Achillea moschata Wulfen, an Endemic Species from the Alps. *Molecules* 21.
